# Microperimetric Biofeedback Training Improved Visual Acuity after Successful Macular Hole Surgery

**DOI:** 10.1155/2015/572942

**Published:** 2015-12-13

**Authors:** Tomoko Ueda-Consolvo, Mitsuya Otsuka, Yumiko Hayashi, Masaaki Ishida, Atsushi Hayashi

**Affiliations:** Department of Ophthalmology, Graduate School of Medicine and Pharmaceutical Sciences, University of Toyama, 2630 Sugitani, Toyama 930-0194, Japan

## Abstract

*Purpose*. To evaluate the efficacy of setting a preferred retinal locus relocation target (PRT) and performing Macular Integrity Assessment (MAIA) biofeedback training in patients showing insufficient recovery of best corrected visual acuity (BCVA) despite successful closure of an idiopathic macular hole (MH).* Methods*. Retrospective interventional case series. Nine eyes of 9 consecutive patients with the decimal BCVA of less than 0.6 at more than 3 months after successful MH surgery were included. A PRT was chosen based on MAIA microperimetry and the patients underwent MAIA biofeedback training. BCVA, reading speed, fixation stability, and 63% bivariate contour ellipse area (BCEA) were evaluated before and after the training. Statistical analysis was carried out using paired Student's *t*-test.* Results*. PRT was chosen on the nasal side of the closed MH fovea in 8 patients. After the MAIA training, BCVA improved in all patients. The mean logMAR value of BCVA significantly improved from 0.33 to 0.12 (*p* = 0.007). Reading speed improved in all patients (*p* = 0.29), fixation stability improved in 5 patients (*p* = 0.70), and 63% BCEA improved in 7 patients (*p* = 0.21), although these improvements were not statistically significant.* Conclusion*. MAIA biofeedback training improved visual acuity in patients with insufficient recovery of BCVA after successful MH surgery.

## 1. Introduction

Vision impairment and metamorphopsia are major symptoms of patients with an idiopathic macular hole (MH). MH surgery has been improved since Kelly and Wendel first reported successful closure of MHs by use of pars plana vitrectomy with gas-fluid exchange [1]. A technique of internal limiting membrane (ILM) peeling improved visual outcomes and the closure rate in MH surgeries [2, 3]. Microincision vitrectomy surgery has shortened the operating time and improved patient comfort and visual recovery time [4]. Numerous studies have reported preoperative predictive factors for visual outcomes following MH surgery, including stage and size of MH [5], duration of symptoms [6], preoperative visual acuity [7], retinal sensitivity, fixation status [8], and optical coherence tomography (OCT) parameters such as minimum diameter of MH, base hole diameter, the hole form factor, MH index, and inner segment/outer segment junction defect length [9]. These factors are not enough, however, to predict visual outcomes precisely. Visual outcomes are often worse than expected despite successful MH surgery.

Recently, foveal displacement following MH surgery has been reported [10–13]. After ILM peeling and gas tamponade, the retina is displaced toward the optic disc [10–13]. Ishida et al. showed that the ratio of retinal displacement in the temporal field was significantly correlated with the basal diameter of the MH [11]. We hypothesized that the foveal displacement might be one of the reasons for poor visual recovery after successful MH surgery. Helping patients to fix at the point of the best visual acuity after the closure of MH might improve their visual performances.

Biofeedback training by means of a microperimeter has been reported as an efficient method to improve the visual performance of patients with macular diseases such as age-related macular degeneration, Stargardt's disease, cone dystrophy, vitelliform dystrophy, and posttraumatic macular scar [14–16]. The training helped the patients stably fix their gaze at the preferred retinal locus relocation target (PRT) near the fovea.

In this study, we performed biofeedback training using a Macular Integrity Assessment (MAIA) microperimeter to provide an efficient PRT and to improve visual performance in patients who had insufficient visual recovery after successful closure of a MH.

## 2. Methods

### 2.1. Inclusion and Exclusion Criteria

We recruited 9 eyes of 9 consecutive patients (4 men and 5 women), who had undergone MH surgery at Toyama University Hospital between May 2013 and June 2014. Inclusion criteria were (1) patients with idiopathic MH, (2) patients in whom closure of MH was confirmed with optical coherence tomography (OCT) by the first MH surgery, (3) patients with visual acuity of 0.6 or less after 3 months of the MH surgery, (4) patients who could undergo OCT examinations with a single spectral domain OCT machine (RS-3000 Advance, NIDEK Co., Ltd., Aichi, Japan) before and after the MH surgery, and (5) patients who agreed with the MAIA training and were followed up for more than 3 months. Exclusion criteria were the presence of ocular complications that could affect visual performance, such as macular degeneration, rhegmatogenous retinal detachment, diabetic retinopathy, glaucoma, and corneal diseases.

### 2.2. Ophthalmic Examinations

All patients underwent comprehensive ophthalmologic examinations, including measurement of the decimal best corrected visual acuity (BCVA), reading speed test, intraocular pressure, slit-lamp biomicroscopy with a contact lens, OCT, and fixation stability test. Axial length was measured in all eyes preoperatively (OA-1000, Tomey, Aichi, Japan). The single spectral domain OCT machine (RS-3000 Advance) was used to evaluate tomographic features through the macula. The macula was scanned with the macula map mode of 9 mm × 9 mm scan (512 × 128) or 9 mm × 12 mm scan (256 × 128) by RS-3000 Advance. The basal diameter of each MH was measured with a caliper built in the software of the OCT machine.

All eyes underwent a standard pars plana vitrectomy with three 25-gauge ports. All patients underwent ILM peeling around the MH in the same manner. Sulfur hexafluoride gas (20%) was used as a tamponade gas at the end of the surgery and the patients were asked to adopt face-down position for at least one hour after the surgery.

**Figure 1:**
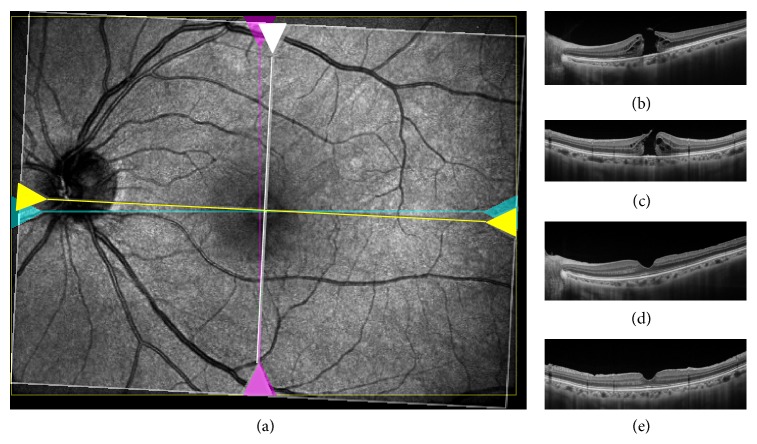
(a) Composite infrared image made by overlapping preoperative and postoperative images (case  6). Yellow line and white line indicate preoperative location of the horizontal and vertical scan (b, c). Blue line and pink line indicate postoperative location of the horizontal and vertical scan (d, e). Center of MH and fovea were identified by moving the location of scans (yellow, white, blue, and pink lines). The fovea has shifted toward the optic disc after MH surgery.

The distance of foveal displacement in each eye was measured after the closure of MH according to the methods of Kawano et al. [10]. Briefly, infrared fundus images were taken together with OCT images. The center of the MH was marked in the preoperative infrared fundus image by referencing OCT images (Figures 1(a), 1(b), and 1(c)), and the length on the image was corrected with the axial length. Postoperatively, the center of foveal depression was similarly marked in the infrared fundus image by reference with OCT images within 3 months after the MH surgery (Figures 1(a), 1(d), and 1(e)). The two marked infrared fundus images were overlapped manually (Figure 1(a)) and the distance of foveal displacement between the marks was measured using ImageJ software (National Institutes of Health, Bethesda, MD, available at http://imagej.nih.gov/ij/).

Reading speed was measured by reading Japanese words written in black on white background at a distance of 30 cm with appropriate refractive correction (MNRead-J, Handaya, Tokyo, Japan). Patients were asked to read the letters aloud as fast as possible without skipping any letters. The sentences contained high-frequency, nontechnical words. A fixation stability test was performed with an MAIA microperimeter (Topcon, Tokyo, Japan). The percentage of fixation points located within the 2-degree circle of the PRT was measured.

Sixty-three percent of bivariate contour ellipse area (BCEA) was also measured to evaluate fixation stability. The 63% BCEA is the elliptical area which encompasses 63% of fixation points during one fixation trial. A smaller BCEA correlates to more stable fixation. Square degree (Sqd) was used as the unit for the 63% BCEA.

### 2.3. Defining Preferred Retinal Locus Relocation Target (PRT)

**Figure 2:**
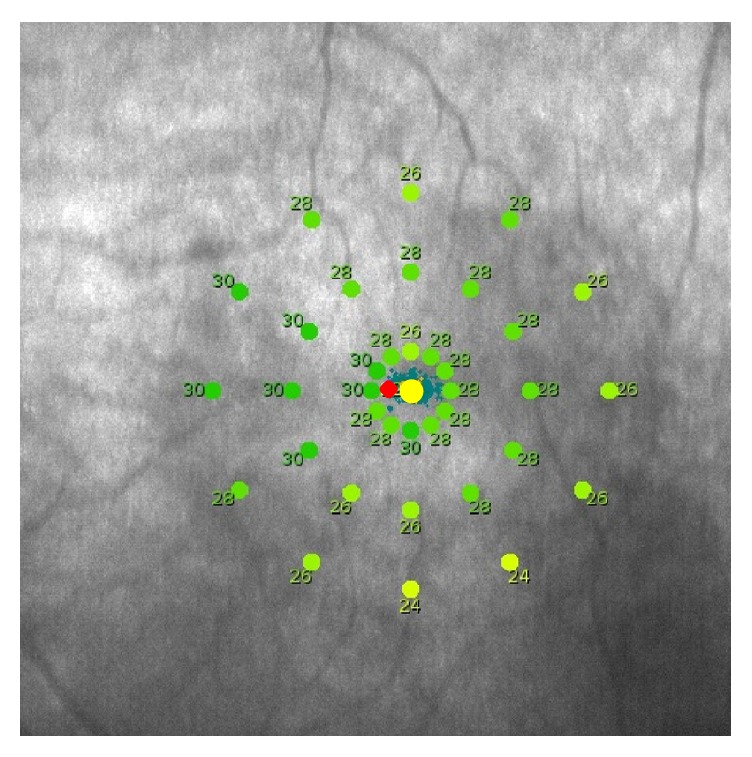
Each colored dot indicates retinal threshold sensitivity (case  8). We chose a region within the 2-degree ring that had the highest potential retinal sensitivity and set the new PRT (red dot) within that area.

Before starting biofeedback training, macular threshold sensitivity and fixation stability were assessed with a MAIA microperimeter using an automated program. Retinal threshold sensitivity was displayed within a 10-degree range of the gravitational center of all fixation points. We chose a region within the 2-degree ring that had the highest potential retinal sensitivity and set the new PRT within that area (Figure 2). If the retinal threshold sensitivity did not exhibit any difference within 2-degree ring, 6 points were screened as candidate PRTs and one point was selected according to fixation stability.

### 2.4. MAIA Biofeedback Training

MAIA biofeedback training was performed using a PRL training module for 10 minutes each session and repeated at least three times within three months. Before beginning the MAIA biofeedback training, the patients were asked to fix their gaze at the PRT by themselves according to an audio feedback program equipped with MAIA. This audio feedback program advised the patients whether or not they were getting closer to the PRT. All the procedures were checked by the examiner.

### 2.5. Statistical Analysis

All statistical analyses were carried out using JMP statistical discovery software (Version 9; SAS Institute, Cary, NC). Paired Student's *t*-test was used to compare the differences between values before and after the training. Spearman correlation coefficient was used to investigate correlations between 63% BCEA and BCVA. Statistical significance was defined as *p* < 0.05. The BCVA was measured with a Landolt C chart in decimal units and converted to a logarithm of the minimum angle of resolution (logMAR) for statistical analysis.

## 3. Results

### 3.1. Characteristics of the Patients

Nine eyes of 9 patients were examined. The preoperative characteristics of the patients are listed in Table 1. The patients' ages ranged from 64 years old to 75 years old (67.2 ± 3.4 years old, mean ± standard deviation (SD)). The decimal BCVA before idiopathic MH surgery ranged from 0.1 to 0.8. There were 8 eyes with stage 3 macular holes and 1 eye with stage 4 macular hole. In the preoperative OCT images, the basal diameter of the macular hole ranged from 369 *μ*m to 949 *μ*m (592 ± 178 *μ*m; mean ± SD). Anatomical closure of the MH was confirmed by OCT examination and no recurrence of MH occurred in any patients in this study. The mean distance of foveal displacement was 155.4 ± 102.6 *μ*m (range: 35.7 *μ*m to 387.6 *μ*m). Eight of the 9 eyes revealed displacement of the foveal center in the nasal direction and in the one eye the foveal center displaced in the temporal direction.

The PRT in each patient was selected according to the sensitivity of the results of microperimetry and the patient's responses. The PRT was selected on the nasal side of the foveal center of the closed MH within 2 degrees in 8 of the 9 patients and in the other patient the PRT was chosen superior to the foveal center of the closed MH within 2 degrees. The patients started MAIA biofeedback training at 3 to 9 months (4.6 ± 1.9 months) after the MH surgery (Table 2). When the patients wished to continue the MAIA biofeedback training after completing 3 sessions, we allowed them to continue it. Five patients ended MAIA training after 3 sessions, while 2 patients performed 4 sessions and 2 patients completed 5 sessions (3.7 ± 0.8 times; mean ± SD). The training period ranged from 1.5 to 4 months (2.3 ± 0.7 months; mean ± SD) (Table 2).

### 3.2. Visual Acuity

**Figure 3:**
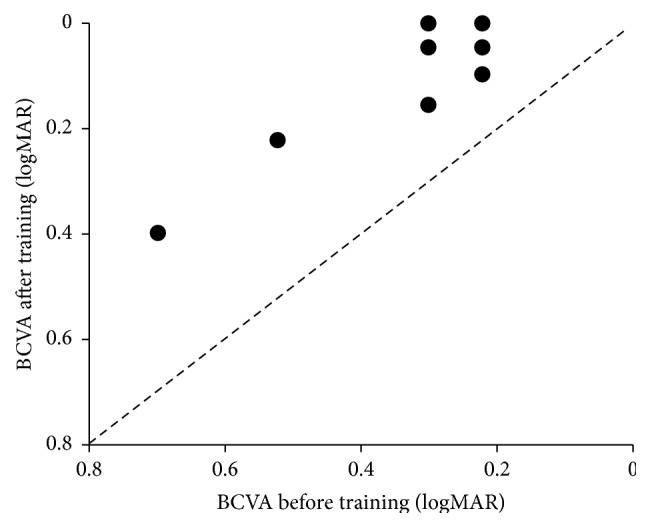
Scatter plot of best corrected visual acuity (BCVA) before and after MAIA biofeedback training.

BCVA improved in all patients after the MAIA biofeedback training (Figure 3). The mean logMAR value of BCVA was 0.33 at the baseline, but it significantly improved to 0.12 after 3 MAIA training sessions (*p* = 0.007) (Table 2). Five patients showed rapid improvement of BCVA after the initial MAIA training.

### 3.3. Reading Speed

The MAIA biofeedback training accelerated the reading speed in all patients. The reading speed before the training was 339 ± 72 words/min and slightly improved to 378 ± 71 words/min at the end of the training, a difference that was not statistically significant (*p* = 0.29) (Table 2).

### 3.4. Fixation Stability

**Figure 4:**
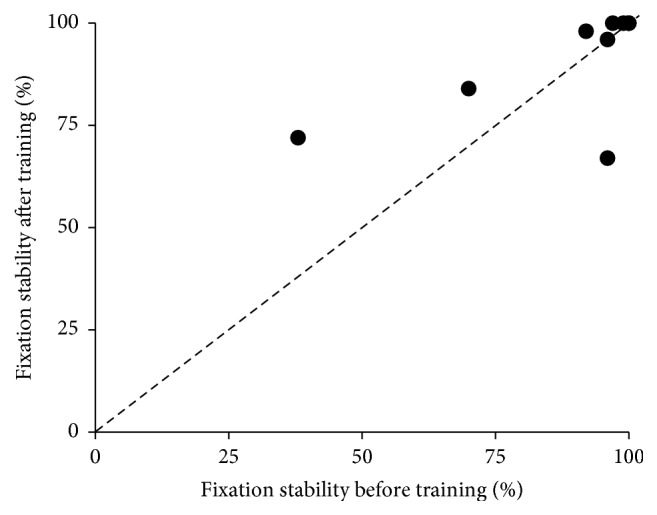
Scatter plot of fixation stability before and after MAIA biofeedback training.

Fixation stability was improved in five patients (patients 1, 2, 4, 5, and 6 in Table 2). In three patients (7–9 in Table 2), fixation stability was unchanged following the training. Two of those patients (patients 8 and 9 in Table 2) showed 100% fixation sensitivity before the start of MAIA training. In one patient (patient 3 in Table 2), fixation stability worsened. The mean fixation stability slightly improved from 88 ± 20% before the training to 91 ± 12% after the training, a difference that was not statistically significant (*p* = 0.70) (Table 2 and Figure 4).

The 63% BCEA was improved in 7 of 9 patients. In one patient the 63% BCEA was unchanged. The 63% BCEA was worsened in one patient. The mean 63% BCEA improved from 0.96 ± 0.91 Sqd before the training to 0.42 ± 0.49 Sqd after the training, although it was not statistically significant (*p* = 0.21) (Table 2). There was no correlation between 63% BCEA and BCVA before the training (Spearman correlation coefficients, *r* = 0.36; *p* = 0.34) or after the training (*r* = 0.27; *p* = 0.49).

## 4. Discussion

The current study demonstrated that biofeedback training using MAIA microperimetry effectively improved the visual acuity of patients when the visual acuity had not fully recovered after successful closure of the MH.

Microperimetric biofeedback training has been performed to improve visual performance of patients with several diseases such as age-related macular degeneration, Stargardt's disease, cone dystrophy, macular myopic degeneration, vitelliform dystrophy, and posttraumatic macular scars [14–16]. Patients with these diseases lost the fixation at the central fovea and were trained to fix at an extra-foveal area (PRT) to improve their visual acuity. In this study, we examined the possibility of biofeedback training to improve the BCVA and visual performances in patients with insufficient recovery of visual acuity after the successful closure of a MH.

One of the advantages using MAIA biofeedback training was that the patients could easily understand and repeat the training with the aid of the audio feedback informing them whether or not they were getting closer to the PRT. Sound perception increases the conscious attention of patients [17], and the increased attention helps the brain fix the PRT [14, 18]. Vingolo et al. reported that audio feedback facilitated stimuli transmission between intraterinal neurons as well as between the retina and the brain and supports a “remapping phenomenon” [15]. The second advantage was that the examiner could select an appropriate PRT in the macula according to the results of the microperimetry and the patients could repeat the training at the same PRT every time.

Kawano et al. reported that the center of the macula area moved toward the optic disc an average distance of 0.1 disc diameter after vitrectomy with ILM peeling [10]. Ishida et al. showed that the ratio of the displacement of the temporal vessel was significantly correlated with the maximum size of the preoperative MH [11]. Nakagomi et al. showed that the postoperative fovea-to-disc distance (3.82 ± 0.34 mm) was significantly shorter than the preoperative one (4.00 ± 0.33 mm, *p* < 0.0001) [13]. These results suggest that the center of the fovea shifted after successful MH surgery with ILM peeling in most patients. Foveal displacement following MH surgery might be one of the reasons for insufficient visual recovery after the closure of MH, and introducing a PRT near the anatomical center of the fovea might result in better visual performance such as visual acuity, reading speed, and fixation stability for those patients. In our study, careful selection of a new PRT, followed by MAIA biofeedback training, improved visual acuity in all patients.

The mean distance of foveal displacement in this study did not differ from those in previous studies [10, 11, 13]. Therefore, patients who showed insufficient recovery of visual acuity were not categorized into a specific group of patients. In addition, the correlation between the location of the PRT and the distance of foveal displacement was unclear. This might be due to the fact that the preferred retinal locus was located on any margin of the preoperative macular hole [19]. In this study, however, the PRT was located slightly to the nasal side of the foveal center in 8 patients (89%). It appears that the fovea with the highest visual acuity might shift to the nasal side of the retina after successful closure of MH.

Visual recovery after macular hole surgery sometimes requires more than 3 months. We cannot exclude the possibility that the visual acuity in our cases improved as part of the natural course during MAIA biofeedback training. In our investigation, however, BCVA immediately improved after the initial MAIA training in 5 of 9 patients and improved further after the completion of 3 MAIA training sessions.

The patients did not show a significant improvement of reading speed, fixation stability, nor 63% BCEA after MAIA biofeedback training. Cappello et al. reported that the maximum reading speed was significantly improved after MH surgery [20]. Another group showed that the mean maximum reading speeds were comparable for eyes with closed MHs and their healthy fellow eyes [21]. Because we examined reading speed, fixation stability, and 63% BCEA of the patients with successful closure of the MH, a significant improvement was not detected even though all patients showed a tendency to improve their visual performance after MAIA biofeedback training. Tarita-Nistor et al. reported that change in fixation stability was a strong predictor of visual outcome after successful closure of the macular hole in 10 patients [22]. As shown in Table 2, patients 1 and 4 improved their fixation stability more than 14% by MAIA biofeedback training.

In patient 3, fixation stability and 63% BCEA worsened after the training. It was possible that the point of PRT became inapt while retinal sensitivity was improved nonuniformly during the training. This case suggested that we should recalibrate PRT even during the training when fixation stability or 63% BCEA worsen.

Limitations of this study are the small sample size and the nonrandomized comparison. The effective number of training session and intervals between each training session are also unknown. However, most of the patients experienced an immediate improvement in visual performance after the first MAIA biofeedback training in this study. MAIA biofeedback training was easy for most patients and could be an option for patients whose recovery of visual acuity after successful MH surgery is insufficient.

Further studies with larger numbers of patients are needed to identify the most effective locations of the PRT and compare different methods of PRT location.

## Figures and Tables

**Table 1:** Preoperative clinical characteristics.

Patient number	Sex	Age (years)	Stage	Duration of symptoms (months)	Basal diameter (*μ*m)	Preoperative decimal BCVA
1	F	64	3	1	571	0.2
2	M	66	3	Unknown	615	0.4
3	F	75	4	2	483	0.8
4	M	70	3	1.5	949	0.15
5	M	69	3	1	457	0.1
6	F	65	3	1	837	0.2
7	F	67	3	2	459	0.15
8	M	64	3	2	588	0.15
9	F	64	3	1	369	0.15

BCVA: best corrected visual acuity.

**Table 2:** Visual performance before and after the training.

Patient number	Period after operation (months)	Training (number of sessions)	Distance of foveal displacement (*µ*m)	Decimal BCVA		Reading speed (words/min)		Fixation stability (%)		63% BCEA (square degree)	
				Pre	Post	Pre	Post	Pre	Post	Pre	Post
1	5	3	89.4	0.2	0.4	243	461	38	72	1.2	0.5
2	3	3	249.0	0.5	0.6	260	292	92	98	0.8	0.2
3	3	3	387.6	0.6	1.0	319	338	96	67	0.3	1.6
4	3	5	35.7	0.3	0.6	279	280	70	84	0.4	0.2
5	6	5	82.7	0.5	1.0	477	486	99	100	0.9	0.1
6	3	4	152.0	0.6	1.0	326	328	97	100	3.3	0.1
7	9	4	192.8	0.5	1.0	387	429	96	96	1.2	0.9
8	4	3	132.7	0.6	0.8	344	360	100	100	0.1	0.1
9	5	3	77.2	0.6	0.8	418	430	100	100	0.4	0.1

BCVA: best corrected visual acuity;

Pre: baseline values;

Post: values at the end of training;

BCEA: bivariate contour ellipse area.
